# County Insane Asylum Affairs

**Published:** 1884-12

**Authors:** S. V. Clevenger

**Affiliations:** Special Pathologist Cook Co. Insane Asylum; Chicago


### DOMESTIC CORRESPONDENCE.

COUNTY INSANE ASYLUM AFFAIRS. *The following communications explain themselves:* AN OPEN LETTER TO THE COMMISSIONERS OF COOK COUNTY, ILLINOIS.

CHICAGO, NOV. 7, 1884.

GENTLEMEN.—At the request of Doctors N. S. Davis, Oscar C. DeWolf, R. L. Rea and other physicians and for strictly medical reasons, I was appointed pathologist of the County Insane Asylum by your honorable Board in May, 1883.

You are aware of the amount of work I have done toward entering up the daily condition of the patients at the Asylum in the case books. The labor justified your giving me a clinical clerk to assist me.

Some of your number, whom I can name, are also aware that the work was made doubly hard for me by hostility on the part of prominent officials there.

You expressed your satisfaction with my efforts to render the treatment of the insane more humane and scientific by reappointing me the following September, 1883.

I was enabled to furnish the medical journals with many articles on the discoveries I had made by personal examination of over one thousand cases and post-mortem work.

Of course medical men are the best judges of the value of that work.

The opposition to my researches increased in proportion as they became more systematized, until finally I found every avenue for learning anything about the patients closed, and had no alternative but to remain in my laboratory.

I was nominated for the Superintendency of the Asylum and had strong assurances of election, but wrote to Commissioner Wasserman, who made the nomination, firmly declining the honor.

Dr. Kiernan was elected Superintendent, and his appointment was hailed in European and American medical circles as the beginning of a better state of things for the insane in America.

I was reappointed Sept., 1884, for the third time, as pathologist.

There seemed a prospect for the medical staff to make a name for itself, as every member was determined to attend strictly to medical matters and raise the standard of the Asylum to something near the reputation of similar institutions in Europe.

You are aware that the Warden, Engineer, and their employés are not in sympathy with the labors of the medical staff.

Your honorable body has been appealed to in various ways to institute a better state of things, but for some reason thus far no attention has been paid to the petitions sent to you.

Numerous threats have been made against the medical staff by the lay officers and employés, and especially against myself, particularly because I voted the Republican ticket at the last presidential election. The night of Wednesday following the election, I was roundly cursed by an employé, who is now defendant in an action for striking a female attendant. Threats were made against me by that person at Mr. Callahan's hotel, near the asylum, and within a few minutes after making these threats a pistol was fired under my bed-room window, the ball breaking the pane and lodging in a book-case near which my wife was standing.

The object of this letter is to state my grievances and to ask you, if under the circumstances, you think best that I should resign because ruffians in the County employ refuse to let me work in peace; whether I should continue my work there while in imminent danger of losing my life; or whether I should be put to pecuniary loss by the stoppage of my salary while unable to continue the scientific work, to perform which I was three times appointed by you; or whether your honorable body can inquire into and remedy such irregularities.

Very respectfully,

S. V. CLEVENGER,

*Special Pathologist Cook Co. Insane Asylum.*

The Doctor says that the Board turned the matter over to the very committee which was responsible for all the trouble and opposition to the physicians.

CHICAGO, NOV. 14, 1884.

*Committee of Public Charities of the Cook County Commissioners:*

Inasmuch as Mr. Leyden and Mr. Hannigan of your committee have made threats against me, and as nearly all the difficulty at the Asylum has arisen from the Committee of Public Charities failing to aid the medical staff and its head in instituting reforms in the Asylum, and it appears plainly that said committee is hostile to the physicians and is carrying on what is known as a "whitewash" investigation, I decline to appear before it, having said all I have to say in my "Open Letter" to the Board.

My wife and son will testify to my room having been shot into by some one at a time when both Mr. Hannigan and James Lee, were on the County farm, and openly boasting of what they proposed to do to the doctors, "particularly Dr. Clevenger."

I have read in the papers of false testimony having outweighed Dr. Kollar's honest statement before you, and expect no better treatment at your hands for any testimony I might bring forward.

S. V. CLEVENGER,
*Special Pathologist Cook Co. Insane Asylum.*

Realizing the inutility of prolonging a battle in which the odds were all against him, the doctor sent in this final communication:

CHICAGO, ILL., Nov. 17, 1884.

*To the Board of Commissioners of Cook County, Ill.*

GENTLEMEN: As the Medical Superintendent of the Cook County Insane Asylum expresses himself satisfied with the issue of the recent "investigation" by the Committee of Public Charities, and as I have no other object in view than to see that the insane in America shall be better taken care of than heretofore, I have the honor of submitting to you the following recommendations:

That my office of pathologist be abolished, and the saving be applied to increasing the salaries of the other physicians.

That medical matters at the Asylum be encouraged, and that politics should be rendered secondary thereto.

That all employés who come in such direct contact with the insane as supervisors and night watchmen, as well as all attendants, should be and remain under the exclusive control of the Medical Superintendent instead of the Warden, as is now the case.

That the assistant physician and the lady physician, who now receive $1,200 and $600 respectively, should be paid as well as the engineer, whose salary is $1,500 per year.

That the clinical clerk, who now is in receipt of only $28 per month, and who has both a classical and medical education, and the druggist, whose salary is $50 per month, should be both paid as well as the storekeeper ($70 per month), and placed in every respect as officials upon an equal footing. Even the cook and baker are better paid than these medical gentlemen.

That the Medical Superintendent be permitted to nominate to your honorable body:

One specialist in the treatment of eye and ear diseases;

One specialist in the treatment of skin diseases;

One specialist in the treatment of female diseases; (a lady physician by preference).

One specialist in the treatment of all cases requiring surgery.

Such specialists to be appointed by you, upon nomination as above mentioned, and to receive no salary for their services. Their actual railroad expenses only to be defrayed by the County, and to visit the Asylum at such times as the Medical Superintendent may suggest. Such specialists also being subject to removal, for proper reasons, by the Medical Superintendent.

That a male and female interne, to serve, each six months, without salary or other emolument, beyond board, washing and lodging, may, at the option of the Medical Superintendent, be appointed.

That the female Supervisor shall be selected from the training school of nurses, upon the recommendation of the Woman's Club of Chicago, but subject to removal, for cause, as other employés, and to be under the orders ot the Medical Superintendent.

I calculate that all these much needed reforms will not increase expenses to the County more than from $500 to $1000

per annum, after deducting the gain through the extinction of my office.

I also believe that your concurrence in these reasonable suggestions will remove the horrible air of secrecy and prevent the recurrence of the inhumanities from which no Asylum is free.

As salary adjustments, for the following fiscal year of the Board, can only be arranged by your honorable body, upon its reorganization, I would respectfully suggest the laying over of full consideration of this communication until after December first, next.

In this, my final communication, I wish to express my sincere thanks to such Commissioners as have heartily coöperated with the medical staff in raising the standard of the Asylum, and to assure such that posterity will remember their names. Very respectfully,

S. V. CLEVENGER,

*Special Pathologist Cook Co. Insane Asylum.*

